# ORMDL3 overexpression facilitates FcεRI‐mediated transcription of proinflammatory cytokines and thapsigargin‐mediated PERK phosphorylation in RBL‐2H3 cells

**DOI:** 10.1002/iid3.489

**Published:** 2021-07-19

**Authors:** Kazuhiro Ogi, Tetsuji Takabayashi, Kaori Tomita, Masafumi Sakashita, Taiyo Morikawa, Takahiro Ninomiya, Masayuki Okamoto, Norihiko Narita, Shigeharu Fujieda

**Affiliations:** ^1^ Division of Otorhinolaryngology Head and Neck Surgery, Department of Sensory and Locomotor Medicine, Faculty of Medical Sciences University of Fukui Fukui Japan

**Keywords:** FTY720, mast cell, ORMDL3, S1P, UPR

## Abstract

**Introduction:**

The chromosomal region 17q21 harbors the human orosomucoid‐like 3 (*ORMDL3*) gene and has been linked to asthma and other inflammatory diseases. ORMDL3 is involved in the unfolded protein response (UPR), lipid metabolism, and inflammatory reactions. We investigated the effects of ORMDL3 overexpression in RBL‐2H3 cells to determine the contribution of ORMDL3 to inflammatory disease development.

**Methods:**

We generated ORMDL3 stably overexpressing RBL‐2H3 cells to assess degranulation, transcriptional upregulation of interleukin‐4 (IL‐4), tumor necrosis factor‐α (TNF‐α), monocyte chemoattractant protein‐1 (MCP‐1), and mitogen‐activated protein kinase (MAPK) phosphorylation via FcεRI. In addition, we examined the effects of ORMDL3 overexpression on thapsigargin (TG)‐mediated proinflammatory cytokine transcription and UPR by monitoring MAPK, protein kinase‐like endoplasmic reticulum kinase (PERK), and inositol‐requiring enzyme 1 (IRE1) phosphorylation.

**Results:**

Overexpression of ORMDL3 enhanced IL‐4, TNF‐α, and MCP‐1 expression after FcεRI cross‐linking, whereas the sphingosine‐1‐phosphate (S1P) agonist FTY720 suppressed this enhancement. There was no significant difference in degranulation and MAPK phosphorylation via FcεRI‐mediated activation between vector‐transfected and ORMDL3‐overexpressing cells. ORMDL3 overexpression accelerated TG‐mediated PERK phosphorylation, while MAPK phosphorylation and proinflammatory cytokine expression showed no significant changes in ORMDL3‐overexpressing cells.

**Conclusions:**

Our findings suggest that ORMDL3 plays an important role in regulating proinflammatory cytokine expression via the S1P pathway and selectively affects the UPR pathway in mast cells.

## Graphical abstract

The messenger RNA (mRNA) expression of TNF‐α, IL‐4, and MCP‐1 were significantly upregulated after engagement of FcεRI in orosomucoid‐like 3 (ORMDL3) overexpressing cells. FTY720 significantly reduced cytokine mRNA expression in ORMDL3‐overexpressing cells and inhibited the expression to a level that was comparable with that observed in vector‐transfected cells.

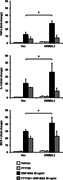

## 1 INTRODUCTION

Orosomucoid‐like 3 (ORMDL3) was first reported to be associated with bronchial asthma and was subsequently reported in various ethnic populations.^1^, ^2^, ^3^, ^4^, ^5^ Recent genome‐wide association studies have linked single‐nucleotide polymorphisms (SNPs) with chromosomal region 17q12‐q21, where human ORMDL3 gene is localized, suggesting the risk of not only asthma but also chronic obstructive pulmonary disease,^6^ Crohn's disease,^7^ inflammatory bowel diseases,^8^ and rheumatoid arthritis.^9^ Significant effort has been put forth to understand the role of ORMDL3 in the development of these inflammatory diseases. We have previously reported that genetic variants in the chromosomal region 17q21 are significantly associated with allergic rhinitis (AR) in the Japanese population.^10^ These are complex diseases that are influenced by multiple genetic and environmental factors. The prevalence of these inflammatory diseases has rapidly increased over the past few decades, and environmental factors have significantly contributed to this increase.^11^ However, the high heritability of allergic diseases also indicates a strong genetic association.^12^, ^13^

Identification of ORMDL3 as a major susceptibility locus by several genome‐wide association studies of human asthma patients suggests that this axis is likely to play an important role in this inflammatory disease. In support of this, ORMDL3 has been shown to be upregulated in asthma.^14^ Our previous study also reported that ORMDL3 is highly expressed in patients harboring the AR susceptibility allele.^10^ ORMDL3 is expressed in multiple cell types, such as epithelial cells, macrophages, eosinophils, and mast cells, and is important in the pathogenesis of inflammatory diseases.^10^, ^15^, ^16^, ^17^ Mast cells were considered to primarily function as effector cells for immediate immunoglobulin E (IgE)‐mediated allergic diseases for decades. However, recent studies imply that mast cells also work as initiator cells in immune surveillance. When activated by pathogens, mast cells provide protection by initiating innate and adaptive immune responses. In support of this, recent studies suggest that mast cells not only play the role of effector cells in allergy but may also function as initiators.^18^ Although mast cells function as important effector cells in IgE‐dependent diseases,^19^, ^20^ the involvement of ORMDL3 on mast cell pathophysiology remains uncertain. RBL‐2H3 cells are derived from a mast cell line cloned from rat leukemia cells, which have been previously utilized for investigating FcεRI cross‐linking and degranulation signal transduction.^21^, ^22^ The same three ORMDL family members, that is, ORMDL1, 2, 3, with ORMDL3 exhibiting high amino acid sequence similarity are expressed in humans and rats.^23^

ORMDL3 belongs to a family of highly conserved transmembrane proteins anchored in the endoplasmic reticulum (ER).^23^ ER stress arises when unfolded/misfolded proteins accumulate in the ER following the disturbance of the ER environment. Cells trigger an adaptive response, the unfolded protein response (UPR), which assists cells to handle the stress. ORMDL3 localizes to the ER and participates in the regulation of the UPR.^24^ UPR relies on three ER stress sensors named inositol‐requiring enzyme‐1 (IRE1), PKR‐like ER kinase (PERK), and activating transcription factor 6 (ATF6). On the contrary, ORMDL3 has been shown to inhibit the sarco/ER calcium pump (SERCA), which subsequently decreases ER Ca^2+^ levels and increases cytosolic Ca^2+^ levels.^24^ It triggers inflammation through activation of NF‐κB and JNK and therefore, leads to inflammatory processes. The ORM protein has also been revealed to be a crucial mediator of sphingolipid homeostasis that might associate with the development of asthma.^25^ ORMDL3 forms a complex with serine palmitoyltransferase, which is involved in the synthesis of the lipid mediator sphingosine‐1‐phosphate (S1P) from sphingosine. S1P induces the release of calcium from intracellular stores activating its receptors S1P receptor‐1 (S1P_1_) and S1P_2_ that are expressed in RBL‐2H3 cells.^26^, ^27^

ORMDL3 may be associated with asthma, AR, and other inflammatory diseases because of its ability to regulate UPR and S1P synthesis. In this study, we investigated the effect of ORMDL3 overexpression on degranulation, proinflammatory cytokine transcription, and the UPR pathway in RBL‐2H3 cells.

## 2 MATERIALS AND METHODS

### 2.1 Construction of plasmid DNA

The complementary DNA (cDNA) encoding human ORMDL3 was amplified from the first‐strand cDNA, which was synthesized using messenger RNA (mRNA) extracted from PC‐3 cells. The primer sequences used for polymerase chain reaction (PCR) were as follows:

5′‐GAGAGGGGCAGCAGGATGAA‐3′ and 5′‐AGGCTTCTTCTTTCTGTCTTCA‐3′. The sequence of the amplified cDNA was verified and ligated into the p3XFLAG‐CMV^TM^‐14 expression vector (Sigma‐Aldrich).

### 2.2 Cell culture and transfection

RBL‐2H3 cell culture was performed as described previously.^28^ For generation of the stable cell line, 5 × 10^6^ RBL‐2H3 cells were transfected with 20 μg of linearized ORMDL3 expression construct using electroporation (950 μF, 310 V). Stably transfected cell lines were selected using culture with 0.4 mg/ml of active G418 (Life Technologies). Generated cell lines were tested for the amount of protein expression using western blot analysis and the whole cell lysates with anti‐ORMDL3 antibody (EMD Millipore Corporation) at 1.0 μg/ml and anti‐β actin antibody (Rockland Immunochemicals Inc.) as internal controls. Two positive cloned lines showing the highest level of ORMDL3 expression were selected for further analysis. For control cells, a linearized empty p3XFLAG‐CMV^TM^‐14 vector was transfected using electroporation.

### 2.3 Analysis of β‐hexosaminidase release

Stably transfected cells (1 × 10^5^) were incubated with anti‐2,4‐dinitrophenol IgE mAb (anti‐DNP IgE, clone SPE‐7; D8406; Sigma‐Aldrich) (1:5000) in 24‐well plates for overnight sensitization. The cells were washed with Tyrode‐HEPES buffer (10 mM HEPES [pH 7.4], 5.6 mM glucose, 0.5 mM KH_2_PO_4_, 10 mM LiCl_2_, 127 mM NaCl, 4 mM KCl, 0.6 mM MgCl_2_, 1 mM CaCl_2_, and 0.1% bovine serum albumin [BSA]) and then stimulated with 1–1000 ng/ml of the DNP‐BSA antigen (LG‐3017; LSL). The activity of released β‐hexosaminidase in the buffer was quantified and shown as described previously.^28^

### 2.4 Quantitative real‐tim**e** polymerase chain reaction (PCR)

Stably transfected cells (1 × 10^6^) were seeded into six‐well plates and incubated with anti‐DNP IgE mAb (1:5000) for overnight sensitization. Sensitized cells were washed with medium to remove unbound IgE and pretreated with 25 μM FTY720 (Sigma‐Aldrich) for 90 min. Thereafter, the cells were washed and activated by DNP‐BSA antigen (30 ng/ml) for 1 h in 37°C incubator. After the stimulation, total RNA was purified using the RNeasy kit (Qiagen) according to manufacturer's instructions. The isolated RNA was reverse‐transcribed to cDNA using the superscript III reverse‐transcriptase (Invitrogen) according to manufacturer's protocol. The quantitative real‐time PCR was conducted using StepOnePlus (Applied Biosystems) and SYBR Premix Ex Taq II (Takara). The primers used in this study were as follows: interleukin (IL‐4) (forward, 5′‐CAGGGTGCTTCGCAAATTTTAC‐3′; reverse, 5′‐ACCGAGAACCCCAGACTTGTT‐3′), tumor necrosis factor‐α (TNF‐α) (forward: 5′‐GTAGCCCACGTCGTAGCAA‐3′; reverse: 5′‐AAATGGCAAATCGGCTGAC‐3′), monocyte chemoattractant protein‐1 (MCP‐1) (forward, 5′‐CGGCTGGAGAACTACAAGAGA‐3′; reverse, 5′‐CTCTTGAGCTTGGTGACAAATACT‐3′), GAPDH (forward: 5′‐TTCACCACCATGGAGAAGGC‐3′; reverse: 5′‐GGCATGGACTGTGGTCATGA‐3′).

### 2.5 Immunoblotting

The stably transfected cells were incubated with anti‐DNP IgE mAb (1:5000) in six‐well plates. The sensitized cell monolayers were stimulated by DNP‐BSA (30 ng/ml) after two washes with Tyrode‐HEPES buffer. When cells were treated with thapsigargin (TG) (Sigma‐Aldrich), they were not pretreated with anti‐DNP IgE mAb. Cell lysis and western blot analysis analyses were performed as described previously.^24^ The anti‐phospho‐extracellular signal‐regulated kinase (ERK) (Thr202/Tyr402), anti‐phospho‐c‐Jun N‐terminal kinase (JNK) (Thr183/Tyr185), anti‐phospho‐PERK (Thr980), and anti‐ERK antibodies were purchased from Cell Signaling Technology; the anit‐phospho‐IRE1 (S724) was purchased from Abcam; and the anti‐JNK antibody was purchased from Millipore. ImageJ (National Institute of Health) was used to quantify the signal, and protein levels were normalized to the corresponding controls.

### 2.6 Statistical analysis

Data were expressed as mean ± standard error of mean (SEM), and all statistical analyses were conducted using statistical software (GraphPad Prism 7). Statistical comparisons were made using unpaired two‐tailed Student's *t* test. *p* < .05 were considered statistically significant.

## 3 RESULTS

### 3.1 ORMDL3 overexpression does not affect degranulation and mitogen‐activated protein kinase (MAPK) phosphorylation via FcεRI in mast cells

**Figure 1:**
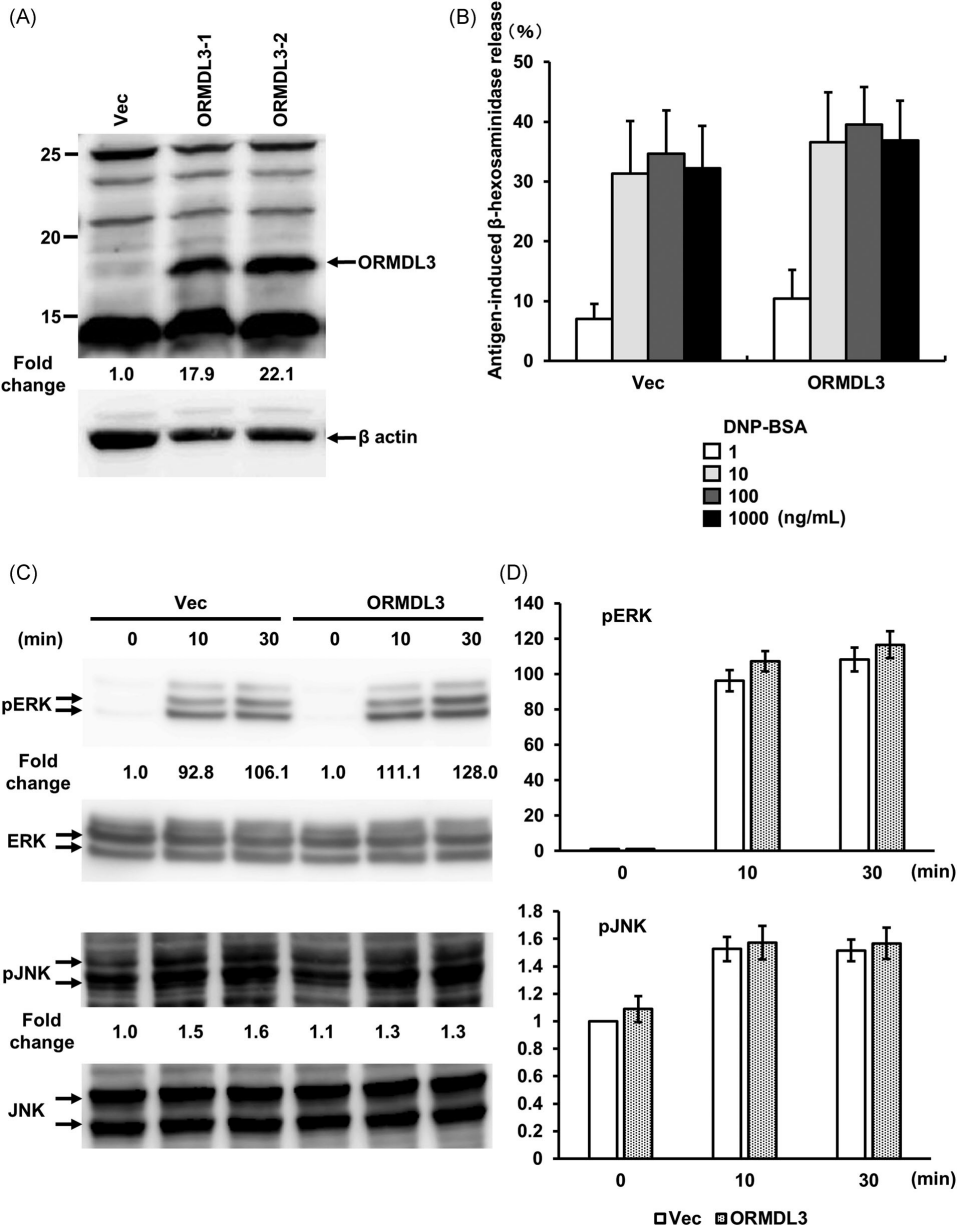
Effect of ORMDL3 overexpression and analysis of antigen‐induced degranulation and MAPK phosphorylation in RBL‐2H3 cells. (A) Expression level of ORMDL3 in vector‐transfected and ORMDL3‐overexpressing RBL‐2H3 cells. The amount of ORMDL3 protein was quantified with rabbit polyclonal anti‐ORMDL3. β‐actin was used as a loading control for normalization. Numbers under the ORMDL3 blot shows ORMDL3 expression level normalized with densitometric intensity of β‐actin (fold change). (B) β‐hexosaminidase release. Each cell line was stimulated by DNP‐BSA antigen at the indicated concentrations after overnight preincubation with anti‐DNP IgE (1:5000). The results are represented as mean values ± SEM from four independent experiments. (C) FcεRI‐mediated ERK and JNK phosphorylation. ERK and JNK were used as references for normalization. Cell lines were sensitized by anti‐DNP IgE (1:5000) and then incubated with 30 ng/ml of DNP‐BSA for 10 and 30 min. Numbers under the immunoblots of PERK and pJNK express the relative amount of PERK and pJNK divided by corresponding ERK and JNK and normalized with its level in vector‐transfected cells after 0 min. Representative immunoblot images from four independent experiments are shown. (D) Quantitative and statistical analysis of ERK and JNK phosphorylation. The normalized densitometric values were shown as fold of increase compared with that in nonactivated vector‐transfected cells (*n* = 4/group). DNP‐BSA, 2,4‐dinitrophenylated‐bovine serum albumin; ERK, extracellular signal‐regulated kinase; IgE, immunoglobulin E; JNK, c‐Jun N‐terminal kinase; MAPK, mitogen‐activated protein kinase; ORMDL3, orosomucoid‐like 3; SEM, standard error of the mean

To analyze ORMDL3 function in mast cells, we used stably transfected RBL‐2H3 cells to overexpress ORMDL3. Densitometric analyses revealed that ORMDL3 transfection led to a 17.9‐22.1‐fold increase in endogenous ORMDL3 protein levels (Figure 1A). We first examined antigen‐induced degranulation by monitoring the β‐hexosaminidase release. Control vector‐transfected cells and cells overexpressing ORMDL3 were sensitized with anti‐DNP IgE mAb and then activated by different concentrations of the DNP‐BSA antigen. There was no significant difference in FcεRI‐mediated degranulation upon overexpression of ORMDL3 (Figure 1B).

Next, we tested the effect of ORMDL3 overexpression on FcεRI‐induced ERK and JNK on MAPK phosphorylation. Similar to the degranulation assay, there was no significant difference in FcεRI‐mediated ERK and JNK phosphorylation between vector‐transfected and ORMDL3‐overexpressing cells (Figures 1C and 1D).

### 3.2 FTY720 inhibits cytokine mRNA expression enhanced by ORMDL3 overexpression in mast cells

**Figure 2:**
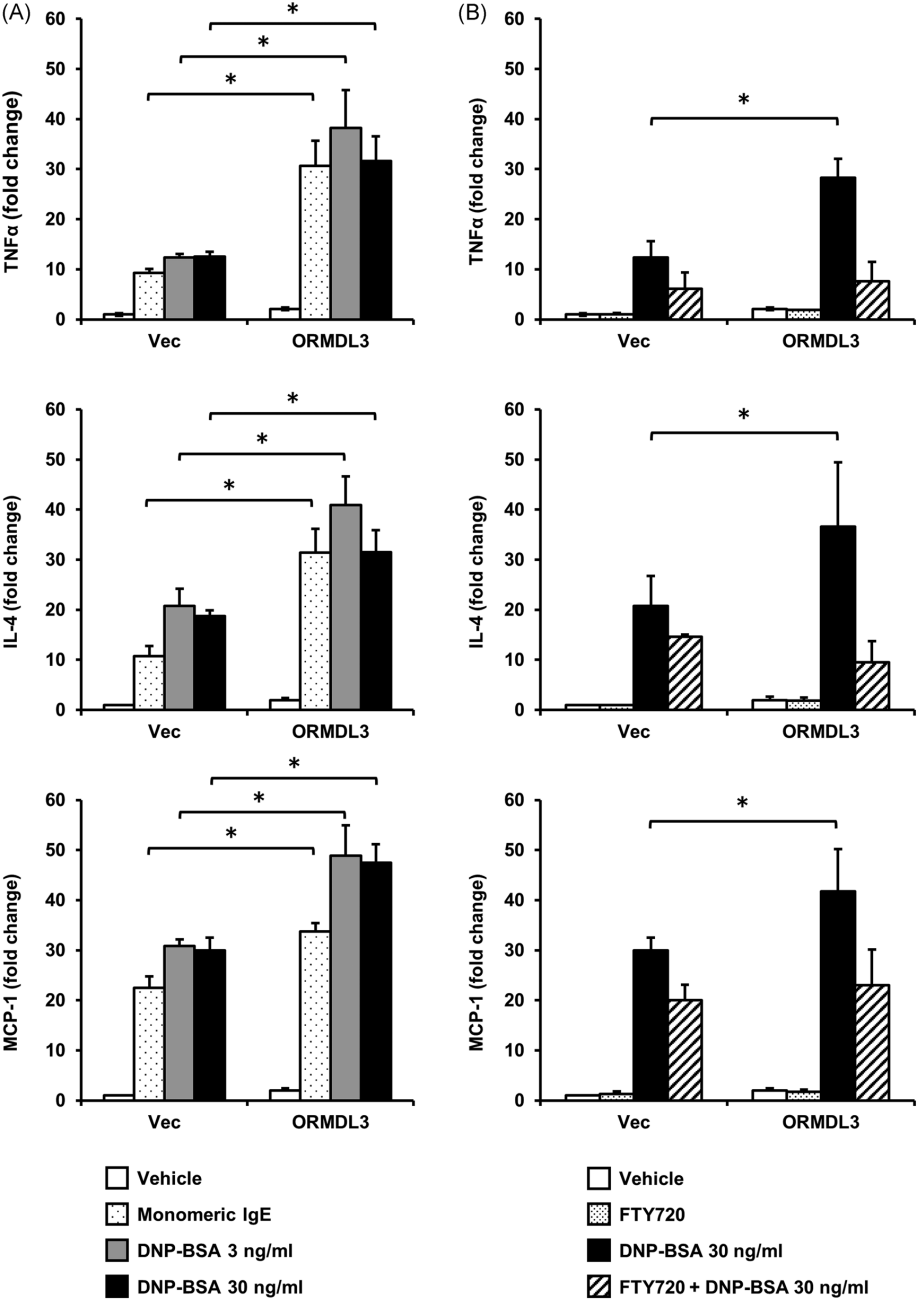
Expression of the proinflammatory cytokines TNF‐α, IL‐4, and MCP‐1 induced by FcεRI cross‐linking in ORMDL3 overexpressing RBL‐2H3 cells. (A) Cell lines were stimulated with 30 ng/ml DNP‐BSA after sensitization for 1 h or monomeric IgE (1:5000) of the same concentration for sensitization. After total RNA extraction and reverse transcription, cDNAs encoding TNF‐α, IL‐4, and MCP‐1 were quantified using real‐time PCR. These expression levels were normalized with housekeeping gene *GAPDH*. (B) Effect of pretreatment with FTY720. Cell lines were pretreated with 25 μM FTY720 for 90 min before stimulation by DNP‐BSA. Data are representative of four independent experiments and are presented as the mean ± SEM. *p < .05 was considered statistically significant (*n* = 4/group). cDNA, DNP‐BSA, 2,4‐dinitrophenylated‐bovine serum albumin; IgE, immunoglobulin E; IL‐4, interleukin‐4; MCP‐1, monocyte chemoattractant protein‐1; ORMDL3, orosomucoid‐like 3; PCR, polymerase chain reaction; SEM, standard error of the mean; TNF‐α, tumor necrosis factor‐α

Next, we tested the effects of ORMDL3 overexpression on mRNA expression of proinflammatory cytokines. Analysis of mRNA expression using real‐time PCR showed that IL‐4, TNF‐α, and MCP‐1 were significantly upregulated after engagement of FcεRI by 3 or 30 ng/ml DNP‐BSA (Figure 2A). The expression of IL‐4, TNF‐α, and MCP‐1 were also enhanced by monomeric IgE stimulation. We tested whether the enhanced cytokine mRNA expression was inhibited by pretreatment with FTY720, an S1P receptor agonist known to induce internalization of the S1P receptor. As expected, FTY720 significantly reduced cytokine mRNA expression in ORMDL3‐overexpressing cells and inhibited the expression to a level that was comparable with that observed in vector‐transfected cells (Figure 2B). These findings demonstrated that the S1P pathway played an important role in the mRNA expression of proinflammatory cytokines in mast cells.

### 3.3 ORMDL3 overexpression selectively alters TG‐mediated PERK phosphorylation kinetics of UPR pathway in mast cells

**Figure 3:**
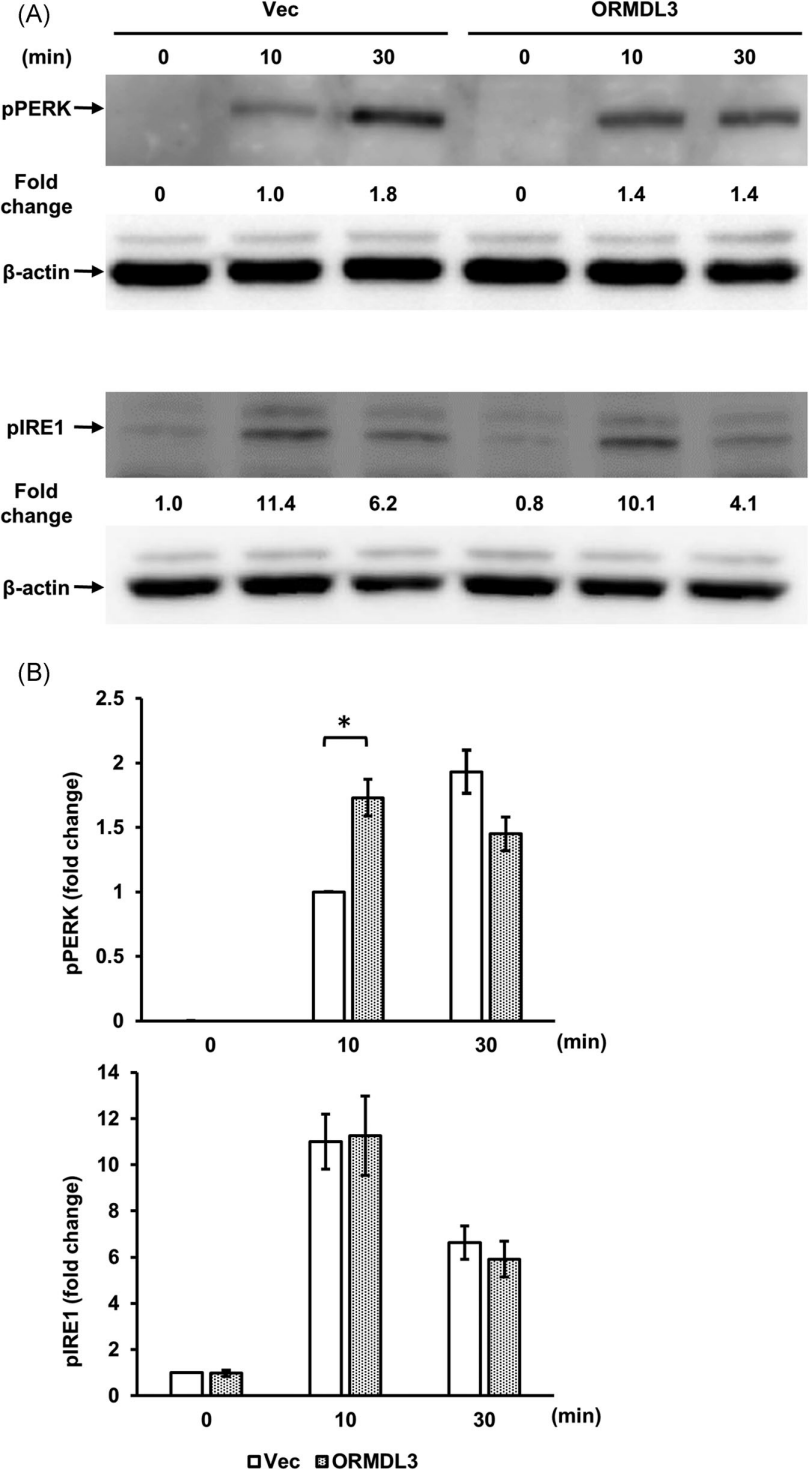
UPR pathway kinetics change in ORMDL3 overexpressing RBL‐2H3 cells. (A) Phospho‐PERK (pPERK) and phospho‐IRE1 (pIRE1) change in ORMDL3 overexpressing RBL‐2H3 cells. Cell lines were incubated with 10 μM TG for 10 and 30 min. β‐actin was used as the loading control. Numbers under the pPERK and pIRE1 immunoblot designate the expression levels of pPERK and pIRE1 divided by corresponding expression level of β‐actin and normalized with its levels in vector‐transfected cells after 10 min (pPERK) or 0 min (pIRE1). Representative immunoblot images from three independent experiments are shown. (B) Quantitative and statistical analysis of pPERK and pIRE1. The normalized densitometric values were shown after normalization compared with that in control vector‐transfected cells after 10 min (pPERK) or 0 min (pIRE1). *p < .05 was considered statistically significant (*n* = 4/group). ORMDL3, orosomucoid‐like 3; TG, thapsigargin; UPR, unfolded protein response

To elucidate if ORMDL3 overexpression affected the UPR pathway, we performed western blots of two kinases that function upstream of UPR, PERK, and IRE1, after the application of the UPR activator TG (10 µM) to vector‐transfected and ORMDL3 overexpressing cells for 10 and 30 min. At 10 min after the application, we found a significant difference on PERK phosphorylation between the cells of both groups; as TG began phosphorylating PERK after 10 and 30 min in a time‐dependent manner in vector‐transfected cells, ORMDL3 overexpression consistently advanced the peak of PERK phosphorylation in the overexpressing cells (Figures 3A and 3B). In contrast, ORMDL3 overexpression exerted no effects on IRE1 phosphorylation. These data suggested that ORMDL3 overexpression selectively accelerated PERK activation in mast cells.

### 3.4 TG does not affect inflammatory cytokine mRNA expression and MAPK phosphorylation in ORMDL3 overexpressing mast cells

**Figure 4:**
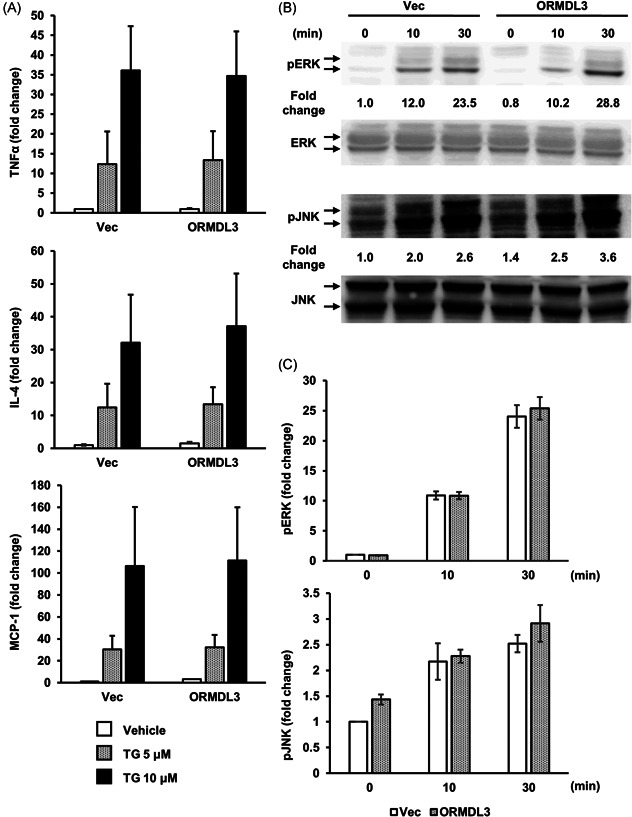
Effects of ORMDL3 overexpression on cytokine mRNA expression after induction of UPR in RBL‐2H3 cells. (A) Cells were treated with 5 or 10 μM thapsigargin (TG), a known activator of the UPR, for 60 min. The expression of cytokines in untreated vector‐transfected cells was used for normalization. Data are representative of four independent experiments and are presented as the mean ± SEM. Western blot analysis of TG‐mediated MAPK phosphorylation (B). ERK and JNK were used as references for normalization. Cell lines were stimulated with 10 μM TG for 10 and 30 min. Numbers under the PERK and pJNK show the expression levels of PERK and pJNK divided by corresponding expression levels of ERK and JNK and normalized with its levels in the vector‐transfected cells after 0 min. Representative immunoblot images from four independent experiments are shown. (C) Quantitative analysis of MAPK phosphorylation. The normalized densitometric values were expressed as fold of increase compared with that in unstimulated vector‐transfected cells (*n* = 4 per group). ERK, extracellular signal‐regulated kinase; JNK, c‐Jun N‐terminal kinase; ORMDL3, orosomucoid‐like 3; MAPK, mitogen‐activated protein kinase; mRNA, messenger RNA; SEM, standard error of the mean; UPR, unfolded protein response

Next, we assessed the effect of ORMDL3 overexpression on the TG‐mediated cytokine mRNA expression and MAPK phosphorylation in RBL‐2H3 cells. TG induces ER stress through MAPK signaling cascades, and MAPK plays a pivotal role in regulation of the UPR.^29^ We examined the expression of IL‐4, TNF‐α, and MCP‐1 after treatment with 5 and 10 µM TG. There was no significant difference in IL‐4, TNF‐α, and MCP‐1 mRNA expression between vector‐transfected and ORMDL3‐overexpressing cells following treatment with 5 and 10 µM TG (Figure 4A). Similarly, there was no significant difference in ERK and JNK phosphorylation between vector‐transfected and ORMDL3‐overexpressing cells following treatment with 10 µM TG (Figure 4B).

## 4 DISCUSSION

The findings of our study demonstrated that overexpression of ORMDL3 facilitated transcription of IL‐4, TNF‐α, and MCP‐1 via FcεRI‐mediated signaling but did not affect MAPK phosphorylation or degranulation in RBL‐2H3 cells. The enhanced transcription of proinflammatory cytokines was suppressed by FTY720. Furthermore, ER stress induced by TG did not significantly affect transcription of these proinflammatory cytokines or MAPK phosphorylation in ORMDL3 overexpressing RBL‐2H3 cells, while the phosphorylation peak of PERK was brought forward. Together, these results suggested that ORMDL3 regulated proinflammatory cytokine production via S1P and selectively regulated the UPR pathway in mast cells. Considering that ORMDL3 is highly expressed in patients with asthma and AR, ORMDL3 is thought to regulate inflammatory cytokine expression positively.^10^, ^14^

FcεRI‐mediated calcium mobilization signaling is one of the most crucial events in mast cells. Previous studies have reported that ORMDL3 regulates the level of cytoplasmic calcium and Ca^2+^ release from the ER.^30^ On the contrary, our data revealed that ORMDL3 overexpression showed no significant difference in FcεRI‐mediated degranulation and MAPK phosphorylation in RBL‐2H3 cells (Figure 1B–D), which require extracellular Ca^2+^ influx. In other words, ORMDL3 overexpression does not affect extracellular Ca^2+^ mobilization via FcεRI in mast cells. Additionally, our unpublished data revealed that ORMDL3 overexpression in the Daudi cells (derived from human B cells) showed no significant difference in B cell receptor‐mediated nuclear factor of activated T cells activation (NFAT) and MAPK phosphorylation, which also require extracellular Ca^2+^ influx (data not shown). In agreement with this finding, a previous study has demonstrated that ORMDL3 is not involved in calcium mobilization in mast cells.^17^ Taken together, ORMDL3 was indicated to play an important role in ER‐mediated intracellular Ca^2+^ release in several studies,^24^, ^30^ but it was not an essential component of FcεRI‐mediated degranulation and MAPK phosphorylation requiring extracellular Ca^2+^ mobilization.

IL‐4 regulates T‐helper type 2 differentiation leading to airway hyper‐responsiveness (AHR).^31^ TNF‐α, a well‐known cytokine, is induced by FcεRI‐mediated signaling, with various functions, including proliferation, angiogenesis, cytotoxicity, and inflammation.^32^ MCP‐1 is a CC chemokine with pathogenetic roles in various diseases; it recruits monocytes to sites of inflammatory responses.^33^, ^34^, ^35^ Although this study had a limitation that the lack of Th1 mediators data including interferon‐gamma, we found that the upregulation of *IL‐4, TNF‐α*, and *MCP‐1* genes by FcεRI‐mediated signaling was enhanced in cells stably overexpressing ORMDL3 (Figure 2A). On the contrary, other study has demonstrated that overexpression of ORMDL3 had no significant effect on cytokine expression in mouse mast cells.^17^ This apparent discrepancy may be associated with differences in the overall ORMDL3 expression, cell types, and experimental conditions. Studies have indicated that ORMDL3 expression varies after particular events, including FcεRI‐mediated activation,^17^ and exposure to poly(I:C),^36^ and house dust mites.^37^ This variable expression toward different stimulation events could explain these contradictory results. In addition, we tested the effect of monomeric IgE as monoclonal IgEs exhibit similar effects induced by IgE cross‐linking with antigen.^38^ Monomeric IgE induced transcription of IL‐4, TNF‐α, and MCP‐1 in vector‐transfected cells, and similar enhancement observed by IgE plus DNP‐BSA was also shown by monomeric IgE in ORMDL3 overexpressing cells (Figure 2A). Given these results, even monomeric IgE enhanced the mRNA expression of inflammatory cytokines in ORMDL3 overexpressing cells; only sensitizing mast cells without exposing them to antigen can result in an inflammatory reaction in patients with high expression of ORMDL3. However, this effect may not be replicated in vivo as a recent study has suggested that this effect only relates to stable trimers of IgE and, thus, is likely to be an artifact of the commercial IgE.^39^ Further knockdown studies with different cell types are necessary to understand the effect of ORMDL3 on cytokine production.

A previous study suggested that increased S1P are likely to be associated with increased AHR in asthma.^40^ FcεRI cross‐linking on RBL‐2H3 cells result in activation of sphingosine kinase (SK) and production of S1P, an alternative second messenger for intracellular Ca^2+^ mobilization. It has been shown that FcεRI chiefly uses a SK‐S1P pathway for Ca^2+^ mobilization.^27^ Our results indicate that the SK‐S1P pathway plays an additional role in intracellular Ca^2+^ mobilization that leads to proinflammatory cytokine production, in addition to conventional phospholipase C/inositol triphosphate pathway. S1P and S1P‐regulating enzymes are known to modulate various pathophysiological processes, including the synthesis of inflammatory mediators, tissue remodeling, and angiogenesis. Therefore, these enzymes may serve as targets for the treatment or prevention of allergic diseases. We pretreated cell lines with 25 µM FTY720 for 90 min because these conditions inhibited sphingolipid synthesis in RBL‐2H3 cells.^41^ FTY720 down regulates S1P_1_ because it can induce irreversible S1P_1_ internalization and degradation.^42^, ^43^, ^44^ FTY720 has been clinically used in patients with multiple sclerosis; thus, our data suggest that it may also be used to treat patients with increased inflammation associated with high ORMDL3 expression in terms of risk alleles for diseases such as asthma and AR.

A previous study has suggested that ORMDL3 plays a crucial role in the development of the UPR.^24^ UPR is activated after unfolded/misfolded proteins accumulate in the ER lumen, thereby triggering PERK phosphorylation and increasing protein folding and protein degradation activity.^45^ TG increases the intracellular Ca^2+^ concentration via inhibition of SERCA,^46^ which results in compulsory extracellular Ca^2+^ mobilization and ER stress. Although it is considered that ORMDL3 inhibits SERCA and subsequently affects UPR, we observed that overexpression of ORMDL3 had no significant effect on the transcription of cytokines or MAPK phosphorylation in RBL‐2H3 cells after treatment with TG (Figures 4A and 4B). Therefore, the extracellular Ca^2+^ mobilization signal triggered by TG is independent of ORMDL3‐mediated SERCA inhibition. PERK is considered to react to an ER stress signal that leads to autophosphorylation through its kinase domain and downstream signaling to the transcriptional apparatus.^47^ Activated IRE1 cleaves XBP‐1 mRNA, which also regulates ER protein folding.^48^ The three branches of UPR (PERK, IRE1, and ATF6) are regulated depending on the persistence of ER stress.^49^ Our data showed that ORMDL3 overexpression accelerated PERK phosphorylation, while the output leading to cytokine expression and MAPK phosphorylation was unaffected. Further studies are required to reveal how the three UPR pathways are affected by ORMDL3.

Our results suggest that selective ORMDL3 suppression is a promising approach to treat intractable inflammatory diseases. However, this approach may be controversial as there are several studies that have contradicted this result based on the expression of ORMDL3. Previous studies revealed that downregulation of ORMDL3 enhance chemotaxis in mast cells,^17^ and selective inhibition of ORMDL3 in airway epithelial cells paradoxically resulted in increased AHR as a consequence of increased S1P level, resulting in increased contractility of airway smooth muscles.^50^ These studies raise concerns about the therapy that inhibits ORMDL3 expression. Therefore, further studies are needed to solve these contradictions.

We demonstrated that overexpression of ORMDL3 facilitated the transcription of the proinflammatory cytokines IL‐4, TNF‐α, and MCP‐1 which was inhibited by FTY720 in RBL‐2H3 cells. It is therefore considered that patients harboring risk SNPs at the chromosomal region 17q12‐q21 are more susceptible to exacerbation of inflammatory disease by proinflammatory mediators. Targeting ORMDL3‐related mechanisms, such as the UPR and lipid metabolism, could provide novel strategies for the treatment of intractable inflammatory diseases.

## CONFLICT OF INTERESTS

The authors declare that there are no conflict of interests.

## AUTHOR CONTRIBUTIONS

Kazuhiro Ogi, Kaori Tomita, and Masafumi Sakashita conducted the biological experiments; Kazuhiro Ogi, Tetsuji Takabayashi, Masayuki Okamoto, and Norihiko Narita designed the study; Kazuhiro Ogi, Taiyo Morikawa, and Takahiro Ninomiya analyzed the data; Kazuhiro Ogi, Tetsuji Takabayashi, and Shigeharu Fujieda drafted the manuscript; all authors critically reviewed the manuscript and approved the final version.

## Data Availability

The data that support the findings of this study are available from the corresponding author upon reasonable request.
